# Whole genome sequence of *Vibrio cholerae* directly from dried spotted filter paper

**DOI:** 10.1371/journal.pntd.0007330

**Published:** 2019-05-30

**Authors:** Angèle H. M. Bénard, Etienne Guenou, Maria Fookes, Jerome Ateudjieu, Watipaso Kasambara, Matthew Siever, Stanislas Rebaudet, Jacques Boncy, Paul Adrien, Renaud Piarroux, David A. Sack, Nicholas Thomson, Amanda K. Debes

**Affiliations:** 1 Wellcome Trust Sanger Institute, Genome campus, Hinxton United Kingdom; 2 M.A. SANTE (Meilleur Accès aux Soins de Santé), Yaoundé, Cameroon; 3 Department of Microbiology and Parasitology, Faculty of Science, University of Buea, Buea, Cameroon; 4 Department of Public Health, Faculty of Medicine and Pharmaceutical Sciences, University of Dschang, Cameroon Dschang Cameroon; 5 Clinical Research Unit, Division of Health Operations Research, Ministry of Public Health, N°8, quartier du Lac (Yaoundé III), Cameroon; 6 Ministry of Health, Lilongwe, Malawi; 7 John Hopkins Bloomberg School of Public Health, Baltimore, Maryland, United States of America; 8 Assistance Publique–Hôpitaux de Marseille (APHM), Marseille, France; 9 Hôpital Européen, Marseille, France; 10 National Laboratory of Public Health in Haiti (LNSP), Ministry of Public Health and Population, Haiti; 11 Directorate for Epidemiology, Laboratory and Research, Ministry of Public Health and Population, Haiti; 12 Sorbonne Université, INSERM, Institut Pierre-Louis d’Epidémiologie et de Santé Publique, APHP, Hôpital Pitié-Salpêtrière, Paris, France; 13 London School of Hygiene and Tropical Medicine, Keppel St, Bloomsbury, London WC1E 7HT, United Kingdom; Liverpool School of Tropical Medicine, UNITED KINGDOM

## Abstract

**Background:**

Global estimates for cholera annually approximate 4 million cases worldwide with 95,000 deaths. Recent outbreaks, including Haiti and Yemen, are reminders that cholera is still a global health concern. Cholera outbreaks can rapidly induce high death tolls by overwhelming the capacity of health facilities, especially in remote areas or areas of civil unrest. Recent studies demonstrated that stool specimens preserved on filter paper facilitate molecular analysis of *Vibrio cholerae* in resource limited settings. Specimens preserved in a rapid, low-cost, safe and sustainable manner for sequencing provides previously unavailable data about circulating cholera strains. This may ultimately contribute new information to shape public policy response on cholera control and elimination.

**Methodology/Principal findings:**

Whole genome sequencing (WGS) recovered close to a complete sequence of the *V*. *cholerae* O1 genome with satisfactory genome coverage from stool specimens enriched in alkaline peptone water (APW) and *V*. *cholerae* culture isolates, both spotted on filter paper. The minimum concentration of *V*. *cholerae* DNA sufficient to produce quality genomic information was 0.02 ng/μL. The genomic data confirmed the presence or absence of genes of epidemiological interest, including cholera toxin and pilus loci. WGS identified a variety of diarrheal pathogens from APW-enriched specimen spotted filter paper, highlighting the potential for this technique to explore the gut microbiome, potentially identifying co-infections, which may impact the severity of disease. WGS demonstrated that these specimens fit within the current global cholera phylogenetic tree, identifying the strains as the 7^th^ pandemic El Tor.

**Conclusions:**

WGS results allowed for mapping of short reads from APW-enriched specimen and culture isolate spotted filter papers. This provided valuable molecular epidemiological sequence information on *V*. *cholerae* strains from remote, low-resource settings. These results identified the presence of co-infecting pathogens while providing rare insight into the specific *V*. *cholerae* strains causing outbreaks in cholera-endemic areas.

## Author summary

Cholera affects more than 4 million people globally every year; people predominantly living in poverty or in resource-constrained conditions including political crises or natural disasters. Cholera’s typical presentation is characterized by rapid onset of acute watery diarrhea and vomiting which can progress from watery stool to shock in as little as four hours. Laboratory conditions needed for culture confirmation and strain preservation are rarely to never present in these affected areas. In fact, many cholera endemic areas in Sub-Saharan African are so remote that even treatment response alone is often challenging. Here we present the genomic analysis of DNA extracted from dried filter paper, which is a low-cost, low-tech and sustainable method. Previously this method has facilitated cholera confirmation by PCR, but we demonstrate that this method is also suitable for whole genome sequencing and subsequent strain characterization by presenting the analysis of samples from an outbreak in a remote area of Cameroon. This method will facilitate the understanding of the molecular epidemiology in cholera-prone areas, which were previously too challenging to attempt. It also introduces a method that can be used on a broader scale for diarrheal disease surveillance, including providing a window into co-infection and microbiome analyses.

## Introduction

Global estimates for moderate to severe diarrhea are estimated to account for 1.6 million deaths annually worldwide and total a burden close to 75 million disability-adjusted life years (DALY) with costs approximating 3.11 billion USD in 2010 [1]. Recent studies have demonstrated the value of whole genome sequencing (WGS) in understanding the molecular evolution and transmission of the etiologic agents that cause moderate to severe diarrhea, including *Vibrio cholerae*. An analysis of > 700 *V*. *cholerae* isolate genomes originating from Asia, Africa, Latin America and the Caribbean spanning a period of more than half a century demonstrated that epidemics of cholera in Africa and the Americas stem from the introduction of a single pandemic lineage from Africa and South Asia [2]. This understanding of cholera was only possible because WGS data provided phylogenetically robust measures of relatedness. This analysis revealed that the currently circulating seventh pandemic of cholera can largely be attributed to a closely related genetic sublineage known as the seventh pandemic El Tor lineage (7PET) that forms a single branch within the diverse *V*. *cholerae* species phylogeny.

Over the past 20 years, WGS has improved our understanding of the molecular evolution and transmission of the etiologic agents that cause moderate to severe diarrhea, including *V*. *cholerae* [3]. Molecular epidemiology has become critical to determine the areas at highest risk for infections, to improve intervention measures such as vaccination campaigns but also to inform the development of new vaccines targeting the appropriate species and genotypes [4–6]. In addition, molecular epidemiological data also provides the information needed to combat the growing threats posed by antimicrobial resistance (AMR) spread [7]. Molecular epidemiology has played an important role in recent years for cholera. The OCV stockpile was created in 2013 and the need to target the doses available to those at greatest risk is important due to limits in vaccine availability[8–10]. Cholera often strikes in remote or resource-poor settings where laboratory capacity is limited, and therefore, it is not always possible to culture specimens from stool. Moreover, the time necessary from specimen collection to isolate preservation can take up to five days, if culture capability is available on site, and up to several weeks if specimens have to be stored on Cary Blair and subsequently transported to a central facility. In addition, specimen storage facilities are not available in every laboratory, and biosafety shipping of infectious strains to specialized genotyping laboratories may be very challenging from cholera-affected countries. Hence, the need for alternative approaches is critical to facilitate access to genomic information from samples collected in all cholera outbreak areas, particularly remote and resource-poor areas.

The primary analysis in this paper is based upon samples collected during a cholera surveillance and response program in the remote area of the Far North Region in Cameroon (FNC), which established and validated the use of dried filter paper for stool specimen preservation [11]. During an outbreak in the fall of 2014, cholera cases were primarily reported from an island in Lake Chad where specimen preservation is generally not possible due to lack of laboratory capacity and lack of cold chain transport. After prior stool enrichment in alkaline peptone water (APW), the enriched specimens were either directly spotted onto filter paper (APW-enriched specimen spotted filter paper) or cultured overnight, from which a single colony was picked and spotted onto filter paper (culture isolate spotted filter paper). The dried spot methodology, with or without prior culture step, was instrumental in providing the ability to preserve genomic material in order to characterize the outbreak. The study demonstrated that dried specimen spotted filter papers can be stored for up to 2 years at room temperature prior to DNA extraction and PCR amplification [12]. Once extracted, the DNA was analysed using multi-locus variable-number tandem repeat analysis (MLVA) and demonstrated that advanced molecular DNA methods could be used on dried specimen spotted filter papers preserved samples [12]. A similar study was performed in 2012 during a cholera epidemic in Sierra Leone, where watery diarrhea was directly sampled on filter paper without prior enrichment in APW, and stored for nearly three years at room temperature before successful MLVA genotyping [13]. Given the limitations of MLVA in providing detailed high-resolution molecular epidemiological features, we sought to determine if the same spotted filter paper material could also be used to perform WGS. This is a proof of principle study demonstrating that DNA extracted from simple, low-cost, APW-enriched and culture isolate spotted filter paper can generate high-quality accurate sequence data that has the potential to inform public health decisions by providing essential information on cholera genotype and co-infection burden.

## Methods

### Ethics statement

APW-enriched specimen and culture isolate spotted filter papers included in the study were isolated from participants enrolled in the “Sustainable Cholera Surveillance for Cameroon” project. The Johns Hopkins Bloomberg School of Public Health Institutional Review Board reviewed and approved this study, IRB No. IRB00003981. Written informed consent was obtained from each participant or their caretaker prior to initiation of study activities.

### Epidemiology and site description

The surveillance methodology, specimen collection, laboratory testing and findings have been previously reported [11,12]. Sixty-five isolates from two distinct outbreaks in the Far North of Cameroon were collected during this time.

### APW-enriched and culture isolate spotted filter paper samples

Cameroonian stool specimens from 65 patients tested positive for *V*. *cholerae* by Crystal VC^TM^ dipstick kit (Arkray Healthcare Pvt Ltd.,Surat, India) and subsequently culture-confirmed. Of these specimens, only 16 were processed according to two different protocols, called hereafter APW-enriched specimen spotted filter paper and culture isolate spotted filter paper.

The specimens referred to as APW-enriched specimen spotted filter papers are derived from stool samples enriched for 6-hours in 1X alkaline peptone water (APW) solution at room temperature. Two drops of the APW-enriched stool specimen were aliquoted onto two circles of a Whatman 903 filter paper card for preservation [12].

The specimens referred to as culture isolate spotted filter paper refers to the same 16 Cameroonian patients’ specimens from which an isolate was able to be cultured. Briefly, the APW-enriched stool specimen was transferred via Cary Blair media to the main health facility for microbiological culture. The specimen was streaked onto TCBS medium overnight at 37°C. From each TCBS culture, a single colony was selected, diluted in 50μL of phosphate-buffered saline (PBS) and aliquoted onto filter paper.

### Viability of *V*. *cholerae* from spotted filter papers

To evaluate spotted filter paper as a new sample preservation method, the viability of *V*. *cholerae* after drying on filter paper blots was tested. *V*. *cholerae* serogroup O1 was grown in liquid culture to a confluence of 1x10^8^CFU/mL; 50μL of bacterial suspension was aliquoted onto Whatman filter paper and allowed to air dry overnight at room temperature (17h). Simultaneously, bacterial suspensions were aliquoted into 4 tubes for *Vibrio* viability experiments: heat-killing, ethanol-killing, bleach-killing and UV-light irradiation were evaluated for their sterilisation potential. After timed incubations with each potential killing agent, the bacterial suspension was spotted onto filter papers and allowed to air-dry overnight. A dried spot was excised from each filter paper and subsequently incubated in APW for 6 hours at 37°C. Following enrichment, specimens were tested via Crystal VC dipstick to assess for any *V*. *cholerae* growth. Concurrently, each specimen was streaked on both TCBS and Luria Agar (LA) plates for overnight culture.

### DNA preparation

For APW-enriched specimen and culture isolate spotted filter papers, a single spot of filter paper was excised at Johns Hopkins facilities, using scissors and inserted into a micro-centrifuge tube, washed twice with 1ml 1X PBS, and boiled with 200μL 1.5% Chelex solution for eight minutes. After a 1-minute centrifugation, the supernatant was transferred to a sterile micro-centrifuge tube. The presence of *V*. *cholerae* was confirmed via multiplex PCRs, first targeting an outer membrane protein, OmpW, in combination with primers targeting cholera toxin A, ctxA. A second PCR confirmed the presence of the rfb gene specific for the O1 serogroup following previously described methods [14,15]. To optimize for WGS following Chelex extraction, the DNA extracted from culture isolate spotted filter papers was subsequently purified by ethanol precipitation as described by Sambrook et al. [16]. The DNA was resuspended with ddH2O and sent for quantification by Qubit 2.0 Fluorometer (Thermofisher) and qPCR (StepOnePlus Real-Time PCR System) before WGS. Only samples with greater than a 0.001 ng/μLμl concentration of *V*. *cholerae* DNA were submitted for WGS.

### WGS generation and analysis

WGS was performed at the Wellcome Trust Sanger Institute on an Illumina HiSeq 2500 platform to generate 100 bp paired-end reads. Short read data are available in the European Nucleotide Archive (ENA) database (S3 Table).

Sequence reads were mapped against reference genome *V*. *cholerae* O1 El Tor reference N16961 (accession numbers LT907989/LT907990) using SMALT v0.7.4 [17]. SMALT was used to index the reference using a k-mer size of 13 and a step size of 6 (-k 13 -s 6) and the reads were aligned using default parameters but with the maximum insert size (i) set as 3 times the mean fragment size of the sequencing library. PCR duplicate reads were identified using Picard v1.92 [18] and flagged as duplicates. A reference-based alignment was obtained by mapping paired-end Illumina reads to DNA sequenced from APW-enriched specimen spotted filter papers and isolate spotted filter papers to the *V*. *cholerae* O1 El Tor reference N16961. Automated annotation was performed using PROKKA v1.11 [19] and a genus-specific database from RefSeq [20].

Variation detection was performed using SamtoolsMpileup v0.1.19 [21] with parameters “-d 1000 -DSugBf” and bcftools v0.1.19 [22] to produce a BCF file of all variant sites. All bases were filtered to remove those with uncertainty in the base call. The bcftools variant quality score was required to be greater than 50 and the mapping quality to be greater than 30. If all reads did not give the same base call, the allele frequency, as calculated by bcftools, was required to be either 0 for bases called the same as the reference, or 1 for bases called as a single nucleotide polymorphism (SNP) (af1 < 0.95). The majority base call was required to be present in at least 75% of reads mapping at the base, (ratio < 0.75), and the minimum mapping depth required was 4 reads, at least two of which had to map to each strand (depth < 4, depth_strand< 2). Finally, strand bias was required to be less than 0.001, map bias less than 0.001 and tail bias less than 0.001. If any of these filters were not met, the base was called as uncertain. A pseudo-genome was constructed by substituting the base call at each site (variant and non-variant) in the BCF file into the reference genome and any site called as uncertain was substituted with an N. Insertions with respect to the reference genome were ignored and deletions with respect to the reference genome were filled with N’s in the pseudo-genome to keep it aligned and the same length as the reference genome used for read mapping. Mapping was visualised with Artemis [23] and ACT [24].

### Assemblies

Short reads from the Cameroonian samples were assembled *de novo* using SPAdes v3.10.0 [25], reordered against the reference sequence with ABACAS [26], and then a metaSPAdes [27] was performed with parameters—meta -t 8 -m 15. Statistics from assemblies were extracted with metaQUAST with parameters—fast -no-check [28]. Genome completeness estimates and checks for contamination were performed using CheckM lineage wf with the following parameters -t 8 -x fa—reduced tree [29]. Kraken v0.10.6 [30] was used to assign taxonomic labels using default parameters and the database Refseq release 72 (27/08/2015). Annotation was performed using the RAST server [31].

### Phylogenetic analysis

A genome distance matrix was obtained by using MASH on SPAdes assemblies as previously described [32][5]. To accurately place our samples into a phylogenetic context, we supplemented our analyses with previously published genomes taken from Weill et al [2]. A Neighbor-Joining tree was generated based on the distance matrix using MASHtree [33]. An outgroup composed of M66, CNRVC960188 and CNRVC961190 was used to reroot the tree using Figtree. The resulting phylogenetic tree and corresponding metadata were visualized using Microreact [34].

## Results

### Stool samples collection, DNA extraction and sequence analysis of *V*. *cholerae* genome from spotted filter papers

Viability tests demonstrated that there was no viable *V*. *cholerae* after drying spotted filter paper overnight (17h), Viability was negative on all specimens evaluated via overnight culture on both TCBS and Luria Agar. This demonstrated that the specimens were no longer infectious for *V*. *cholerae* allowing safe shipping regarding biological risks.

Sixteen *V*. *cholerae* O1 positive specimen pairs were included in this study, comparing APW-enriched specimen spotted filter paper to culture isolate spotted filter paper, both derived from the same original stool specimen. As measured by RT PCR, total DNA concentration was on average 3 times higher when recovered from APW-enriched specimen spotted filter paper than from culture isolate spotted filter paper. Conversely the concentration of *V*. *cholerae* specific DNA was nearly 2.5 times higher from the culture isolate spotted filter paper compared to the APW-enriched specimen spotted on filter paper, as per the median and the mean reported in Table 1. In both cases, the quantity of DNA was sufficient to perform WGS (Table 1). Only two APW-enriched specimen spotted samples, 600064 and 600068, displayed higher *V*. *cholerae* DNA concentration than their respective culture isolate counterpart.

**Table 1:** *V*. *cholerae* enriched and isolate filter paper specimens and DNA quantity.

Sample Name	Sample Type	Quantity Mean of *V*.*cholerae* (pg/ul)	Quantity Mean of Total DNA (pg/ul)
500289	Enriched	1.49	340
500289	Isolate	11.12	130
500291	Enriched	9.48	760
500291	Isolate	13.13	190
600052	Enriched	11.22	1400
600052	Isolate	41.87	480
600055	Enriched	0.10	1130
600055	Isolate	12.79	380
600057	Enriched	6.13	760
600057	Isolate	33.29	1510
600058	Enriched	20.31	1520
600058	Isolate	46.42	580
600059	Enriched	18.94	4490
600059	Isolate	30.50	130
600060	Enriched	13.09	700
600060	Isolate	34.73	300
600061	Enriched	17.80	3040
600061	Isolate	46.14	250
600064	Enriched	28.94	740
600064	Isolate	26.29	350
600065	Enriched	1.10	1120
600065	Isolate	25.69	160
600066	Enriched	2.15	1060
600066	Isolate	4.54	350
600067	Enriched	27.95	1230
600067	Isolate	38.04	220
600068	Enriched	19.85	1290
600068	Isolate	10.80	940
600069	Enriched	3.48	890
600069	Isolate	55.58	110
600071	Enriched	6.07	1770
600071	Isolate	19.49	820
Median	Enriched	10.35	1125
Mean	Enriched	11.76	1390
Median	Isolates	28.4	325
Mean	Isolates	28.15	431.25

**Fig 1:**
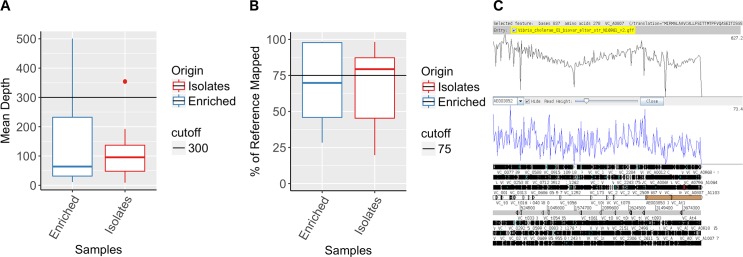
SMALT Mapping of short Illumina reads obtained from sequencing of DNA recovered from Whatman 903 filter cards. Average mean depth obtained from mapping short read Illumina sequences of DNA recovered from Whatman 903 cards (A). Percentage of *V*. *cholerae* reference genome N16961 covered by short Illumina reads mapped by SMALT (B). Artemis visualization of the short Illumina reads of sample 600066 mapped to the *Vibrio cholerae* reference genome N16961 (C).

The mapping coverage for all samples ranged from 8.8x to 500x with an average of 128.4x (Fig 1A). The percentage of *V*. *cholerae* N16961 reference genome covered by reads ranged from 19.71% to 98.33% with an average of 68.44% (Fig 1B).

In both APW-enriched specimen spotted filter papers and culture isolate spotted filter papers, Spearman correlation test showed a positive correlation between the quantity of *V*. *cholerae* DNA and the percentage of *V*. *cholerae* genome covered by short reads in DNA extracted from both APW-enriched and culture isolate spotted filter papers (Spearman correlation score = 0.42, p < 0.1) (S2 Fig). A minimum concentration of 0.02 ng/μL of *V*. *cholerae* specific DNA in the sample generated more than 75% coverage when mapped against the reference genome. 43% of all spotted filter papers that showed successful mapping contained a concentration of *V*. *cholerae* DNA greater than 0.02 ng/μL. The percentage of the reference genome mapped appeared to plateau between a concentration of 0.2 and 0.3 ng/μL.

When comparing the mapping data according to the sample preparation protocol used, APW-enriched specimen spotted filter papers generated slightly higher mapping quality scores, specifically 148.7 ±144.03 mean depth and 70.52% ±26.81 reference genome covered compared to 105.86 ±81.4 mean depth and 66.14% ±25.52 reference genome covered for isolate spotted filter papers (Fig 1). The two protocols, APW-enriched specimen and culture isolate spotted filter paper were not statistically significant (Wilcoxon rank test, p-value = 0.5518 and p-value = 0.1965 for mean depth and reference genome covered respectively).

*De novo* assemblies using SPAdes produced assemblies with < 1000 contigs for 13 out of 16 isolate DNA samples and 6 out of the 16 APW-enriched specimen spotted filter papers. The best assembly with less than 100 contigs including some contigs larger than 50000bp and covering more than 97% of the genome was obtained from DNA extracted from the APW- enriched specimen spotted filter paper for specimen 600057 (Table S3).

**Fig 2:**
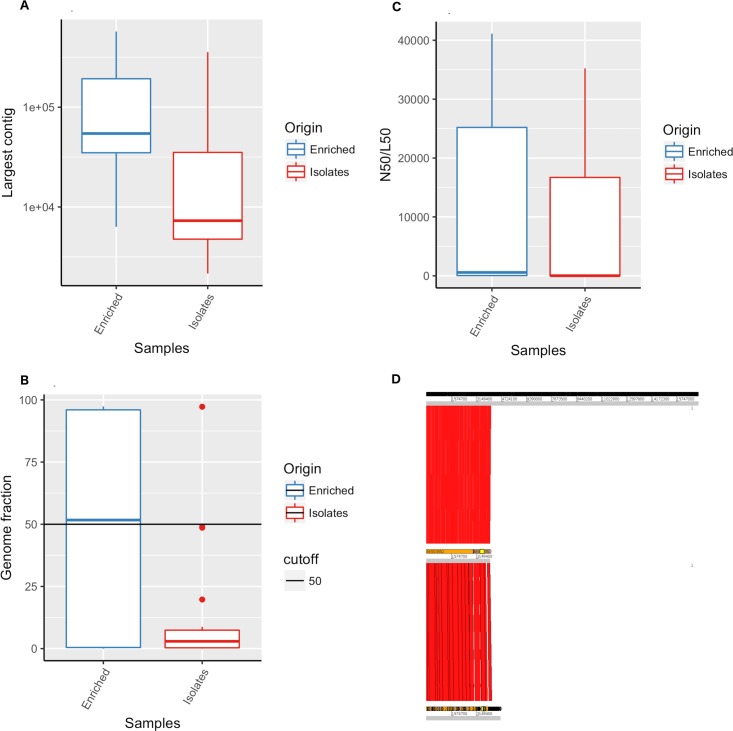
Assembly of genomes obtained from sequencing of DNA recovered from Whatman 903 filter paper cards spotted with APW-enriched specimens and culture derived isolates from patients infected with *Vibrio cholerae* O1. *De novo* spade assembly quality assessment obtained from short read Illumina sequences of DNA recovered from Whatman 903 filter paper cards. Largest contig (A), N50 (B) and percentage of genome fraction (C) of reference genome N16961 covered by metaSPAde assemblies from APW-enriched specimen spotted filter papers versus culture isolate spotted filter papers. ACT comparison of SPAde assemblies obtained from short read Illumina sequences of DNA recovered from Whatman 903 filter paper cards of APW-enriched specimen versus culture isolate spotted filter papers (D).

APW-enriched specimen spotted filter paper exhibited larger contigs than culture isolate spotted filter paper assemblies (Fig 2A). The range in results was higher for the APW-enriched specimen spotted filter papers compared to the culture isolate spotted filter paper, as illustrated by the wide range of reference genome fraction covered by the assemblies of APW-enriched specimen spotted filter papers, varying from 0.007% to 97% (Fig 2B).

MetaSPAde generated assemblies for 15 of the 16 APW-enriched specimen spotted filter papers and 16 out of 16 culture isolate spotted filter papers, while SPAde only produced 24 assemblies out of 32 samples. One APW-enriched specimen sample, 500291, did not produce an assembly from either MetaSPAde analysis or SPAde analysis, likely due to limitations in the quantity of cholera-specific DNA available in the specimen. MetaSPAde analysis of APW-enriched specimen spotted filter papers allowed for the identification of the *V*. *cholerae* genome through sequence assemblies in a higher proportion of samples compared to culture isolate spotted filter papers.

**Fig 3:**
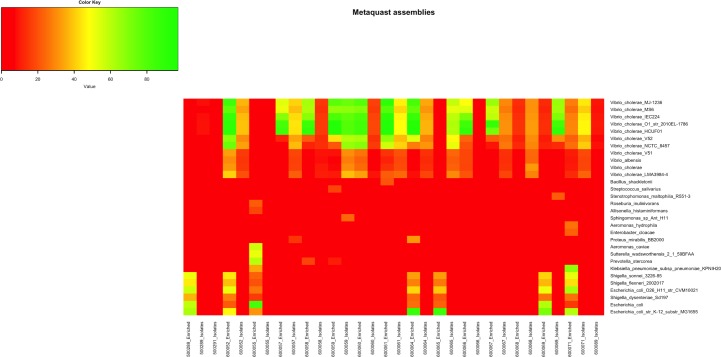
MetaSPAde assemblies of genomes obtained from sequencing of DNA recovered from Whatman 903 filter paper cards. Genomes fraction covered by metaSPAde genome assemblies obtained from short read Illumina sequences of DNA extracted from APW-enriched specimen spotted filter paper and culture isolate spotted filter papers.

Preliminary species diversity analyses of both APW-enriched specimen spotted filter papers and culture isolate spotted filter papers showed that *V*. *cholerae* reads represented one of the most abundant species in the majority of the samples (S3 Fig). Metagenomic analysis of APW-enriched specimen spotted filter paper showed the presence of a diverse microbial population (S3 Fig). As an example, bacteria belonging to the Enterobacteriaceae family such as various *Shigella* species or *Escherichia coli* strains, in addition to *V*. *cholerae* infection were identified in APW-enriched spotted filter papers. The use of MetaSPAde and MetaQUAST software allowed us to determine with higher accuracy the specific contribution of *V*. *cholerae* genome to the assemblies as well as the extent of bacterial diversity found in these samples (Fig 3). As expected, genomic diversity appears higher in enriched specimen spotted filter papers compared to culture isolate spotted filter papers. Similarly, *V*. *cholerae* genome is present in higher proportion in enriched specimen spotted filter papers compared to culture isolate spotted filter papers, which can be easily explained due to the lower quantity of DNA present in the isolate spotted filter papers (Fig 3).

### Whole genome and phylogenetic analysis of *V*. *cholerae* genomes

Of the 32 APW-enriched specimen and culture isolate spotted filter papers samples sequenced, 20 failed to provide coverage above 50% or generate assemblies of greater than 1000 contigs. Therefore, these samples did not meet the criteria for further analysis and were excluded. Assemblies from both the APW-enriched specimen and culture isolate spotted filter papers samples of the same patient were simultaneously aligned with the *V*. *cholerae* reference genome N16961. Alignment comparison demonstrated that assemblies of all APW-enriched specimen spotted filter papers contained contigs outside of the *V*. *cholerae* genome and include sequences that show a high degree of similarity with other bacterial genomes (Fig 3).

The substantial level of coverage facilitated the alignment of short reads to *V*. *cholerae* O1 reference genome, confirming the molecular epidemiological characterization of these strains as *V*. *cholerae* O1. Further, several biologically relevant genes and genomic features of the *V*. *cholerae* genome could be identified in APW-enriched specimen spotted filter papers as well as in culture isolate spotted filter papers. Such examples of genes are *ctxA* and *ctxB* cholera toxin-encoded genes embedded into the integrated CTXΦ prophage; and *tcpA*, a gene of the *Vibrio* pathogenicity island. The strains were not part of O139 serogroup, demonstrated by the absence of genes including *rstA*, or *wfbA* of the *rfb* region (Fig 1C and S4 Fig)[35–38][35–38][34–37].

Variant calling was performed following SMALT mapping in order to identify single nucleotide variant sites (SNV) and sequence diversity within the *V*. *cholerae* genomes of each sample. Variant calling was not performed in samples with a DNA concentration below 0.02 ng/μL (S5A Fig).

**Fig 4:**
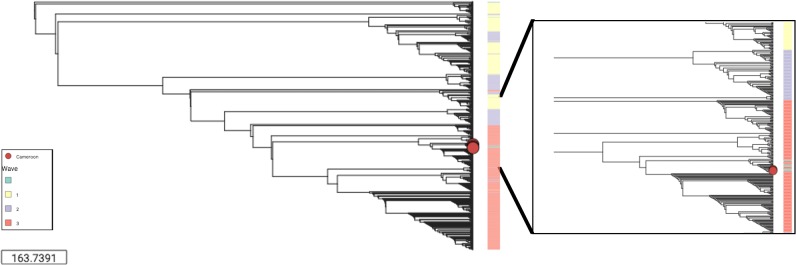
Neighbor-Joining tree representing MASH generated distance matrix based on high quality Spade assemblies.

Compiling all analyses, the highest quality samples were selected based on assembly criteria such as *V*. *cholerae* genome fraction covered (> 50%), number of contigs (> 100), largest contig (> 5000), total assembly length (> 2.2Mb) and NG50 > 500 (S3 Table). Genome distance subsequently estimated using MASH on the eight best spade assemblies of this study were analysed in the context of sequences published by Weill et al. representing *V*. *cholera*e samples spanning over the past century [2]. This data was clustered using a Neighbour-Joining tree demonstrating the characteristic waves reflective of the global phylogeny of the 7^th^ pandemic as shown in (Fig 4) and https://microreact.org/project/S1OfV91PG. The phylogenetic analysis confirmed the affiliation of the Cameroonian *V*. *cholerae* strains extracted from APW-enriched and culture isolate spotted filter papers to the 7^th^ pandemic El Tor. Importantly, the samples fit within the third wave of the global phylogenetic tree of *V*. *cholerae*, in close proximity to other Cameroonian samples of recent years such as those dated from 2005, and from 2010 and 2011.

Based on these observations, we have concluded that the following quality criteria need to be fulfilled for correct phylogenetic interpretation, namely a proportion of reference genome covering greater than 50%, a mean depth greater than 20x, *V*. *cholerae* DNA concentration greater than 0.02 ng/μL, and examination of assemblies (NG50).

## Discussion

In this study, we successfully sequenced DNA from two types of samples spotted onto filter papers for preservation. Not only were these preservation methods proven safe but effective for WGS quality standards after collection, storage and transport.

DNA was recovered from all Cameroonian spotted filter papers and WGS proved to be successful for all samples despite low quantity of DNA recovered. WGS results allowed for mapping short reads for the majority of APW-enriched specimen spotted filter papers and all but one of the culture isolate spotted filter papers. Despite high heterogeneity, the quality of the mapping for APW-enriched specimen spotted filter papers, when compared to culture isolate spotted filter papers, proved to be of variable but satisfactory quality. Quality was illustrated by several criteria including mean depth, proportion of reference genome covered, DNA concentration, and NG50. Mapping confirmed the identification of specific virulence genes and the absence of genes implicated in important biological pathways of *V*. *cholerae*, providing critical molecular epidemiological information to characterize cholera outbreaks in remote and/or unstable areas [39–41][38–40][39–41][35–37]. Furthermore, successful assemblies obtained from WGS of these samples were instrumental in identifying gene context and gene organisation within reconstituted genomes. Analyses suggested that the use of APW enrichment of stool rather than the more laborious selection of isolate spotted filter paper might be more efficient for reconstitution of *V*. *cholerae* assemblies. This data provides a source of information to develop informed experimental hypotheses that may reveal new biological mechanisms of *V*. *cholerae* bacteria.

The use of metagenomics software tools showed bacterial diversity in *V*. *cholerae* infected samples and highlighted the prospect for using spotted filter paper for routine metagenomics analysis. This finding highlights the presence of co-occurrences of potential gut bacterial colonisation or co-infection with other diarrheal pathogens. Novel biological interaction mechanisms may also be explored at the bacterial population level, such as the complexity of the gut microbiota in cholera infected patients [42]. Extracting DNA from APW-enriched specimen spotted filter papers revealed the potential for studying multiple bacterial populations through WGS. The ability to study the diversity of bacterial populations from spotted filter papers will facilitate the study and understanding of the microbiome in low-resource settings, not only as it pertains to cholera.

Mapping based on quality criteria such as the proportion of reference genome covered, mean depth, original concentration of DNA, and high-quality assembly criteria such as NG50, number and size of contigs allowed us to restrict our analysis to high quality assemblies only. This high-quality assembly data could be used to understand the genetic distance between *V*. *cholerae* strains and place the analyzed sample within a general phylogenetic context in the global history of cholera transmission. Pairwise mutation distance-based clustering results confirmed the low level of diversity expected in *V*. *cholerae* clinical samples of an epidemic outbreak. Phylogeny is a critical tool that has been proven to contribute to characterizing outbreaks and to provide evidence for global and local transmission. This tool will be of specific value in remote and resource-constrained settings such as regions where cholera is endemic or regions with elevated risk of cholera epidemics. It will facilitate testing and verification of experimental hypotheses related to the biology of *V*. *cholerae* in controlled laboratory settings where the opportunity may not be otherwise possible. This is a result of difficulties in specimen processing and preservation in remote and austere settings where cholera is often endemic. However, with the increasing affordability of sequencing and the recent development of affordable and compact sequencing technologies, such as ISeq and Oxford Nanopore MinION, access to these technologies in countries at high-risk for cholera is increasing. Together the use of dried specimens in combination with more affordable resources in country may facilitate informed decision-making for a timely response to cholera outbreaks in remote and low-resource areas.

There are several areas of improvement to be considered in future studies. First, since sequencing was not the original intention of the specimen preservation, we did not preserve non-enriched/direct stool samples on the filter paper. Currently, we are working to collect crude, APW-enriched specimen and culture isolate spotted filter papers in tandem to facilitate the comparison of DNA quality as well as sequence results across all potential specimen preservation types to optimize the method most applicable in a low resource setting. Second, the DNA extraction method is an important limitation in the comparison of APW-enriched versus isolate filter paper sequences in this paper. The use of ethanol precipitation likely greatly reduced the final DNA concentration available for sequencing from the isolate spotted filter paper, therefore a direct extraction comparison is warranted. Efforts are currently underway to actively improve the quality and quantity of DNA extracted from filter paper cards through protocol refinement for all specimen types. Subsequent to this study, we have also employed duplicated spotting of specimens at all of our study sites as it has shown to be advantageous providing additional specimen available at minimal cost for these advanced molecular studies. Finally, the wide array of filter paper technologies available will present options for further consideration to determine the specific type of filter paper best for use in subsequent work to optimize DNA preservation on filter paper.

In conclusion, we present a proof of concept for WGS of DNA extracted from APW-enriched specimen and culture isolate spotted filter papers specifically targeted, but not limited, to *V*. *cholerae* strains preserved on dried filter papers. We have determined the minimum methodological requirements allowing for successful WGS that would allow for the retrieval of biologically relevant genomic information. Until sequencing is widely accessible and affordable, the optimization of this method provides high-level molecular information at low cost and limited difficulty to countries at-risk. In conjunction with new sequencing technologies that may soon be available in low-resource settings, we may soon understand transmission patterns in-real time rather than post-outbreak characterization. The optimization of filter paper preservation for WGS will pave the way towards a better understanding of *V*. *cholerae* transmission and outbreak dynamics globally.

## Supporting information

S1 Fig Protocol summary of *Vibrio cholerae* DNA harvest from specimens preserved on filter paper cards. (JPEG)

S2 Fig The quantity of DNA recovered from Whatman 903 filter cards spotted with APW-enriched specimens and culture isolates from patients infected with *Vibrio cholerae* O1 is critical for the quality of mapping. Positive Spearman correlation between the quantity of *Vibrio cholerae* DNA in the material recovered from the Whatman 903 filter cards and the mapping quality as measured by percentage of *Vibrio cholerae* reference genome N16961 covered by short Illumina reads mapped by SMALT and mean depth of short Illumina reads.
(TIF)

S3 Fig Metagenomic analysis of DNA sequences recovered from Whatman 903 filter cards spotted with APW-enriched specimens and culture derived isolates from patients infected with *Vibrio cholerae* O1. Proportion of reads specific to one species over all reads obtained from Kraken analysis of short read Illumina sequences of DNA recovered from Whatman 903 filter cards of APW-enriched specimen spotted filter papers versus culture isolates spotted filter papers (Selective threshold > 0.1%)
(TIF)

S4 Fig Artemis visualization of the short Illumina reads mapped to the *Vibrio cholerae* reference genome N16961 at the *TcpA* gene locus and at *RstA* gene locus. (TIF)

S5 Fig SNP calling from mapping Illumina short reads to reference genome N16961 from DNA recovered from Whatman 903 filter cards spotted with APW-enriched specimens and culture derived Isolates from patients infected with *Vibrio cholerae* O1. SNP calling based on SMALT mapping of short read Illumina sequences of DNA recovered from Whatman 903 filter cards of APW-enriched specimen versus culture isolate spotted filter papers samples (A). Comparison of SNP between APW-enriched specimen and culture isolate among samples with higher than 75% of *Vibrio cholerae* reference genome N16961 mapped(B) and among samples with higher than 50% of *Vibrio cholerae* reference genome N16961 mapped, higher than 0.02ng/μL *Vibrio cholerae* DNA and higher than 20x mean depth (C).
(TIF)

S1 Table SMALT mapping statistics of short Illumina reads obtained from sequencing of DNA recovered from APW-enriched specimen and culture isolate spotted Whatman 903 filter papers. (DOCX)

S2 Table SPAde assembly Quast results of short Illumina reads obtained from sequencing of DNA recovered from APW-enriched specimen and culture isolate spotted Whatman 903 filter papers. (DOCX)

S3 Table Accession numbers of samples used in this study. All data is available at https://www.ebi.ac.uk/ena.
(DOCX)
