# Supplementary figures and images for: Whole genome sequence of Vibrio cholerae directly from dried spotted filter paper
Source: PLoS Negl Trop Dis. 2019 May 30;13(5):e0007330. doi: 10.1371/journal.pntd.0007330 (PMC6559667; doi:10.1371/journal.pntd.0007330)

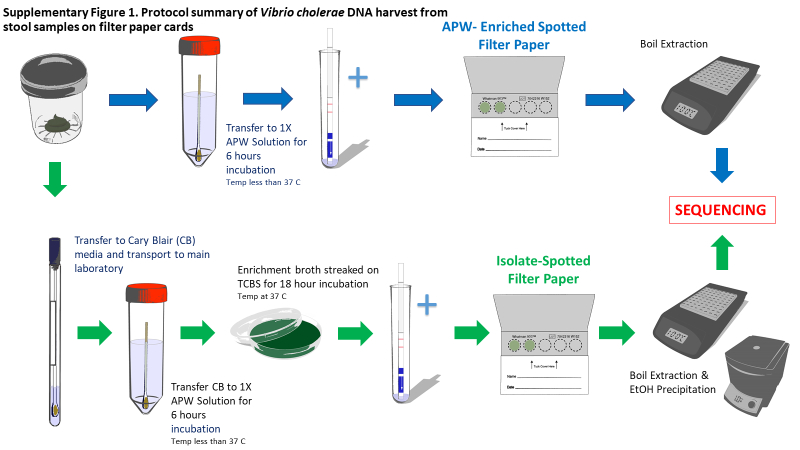

Supplement: S1 Fig — (JPEG) [file pntd.0007330.s001.jpeg]

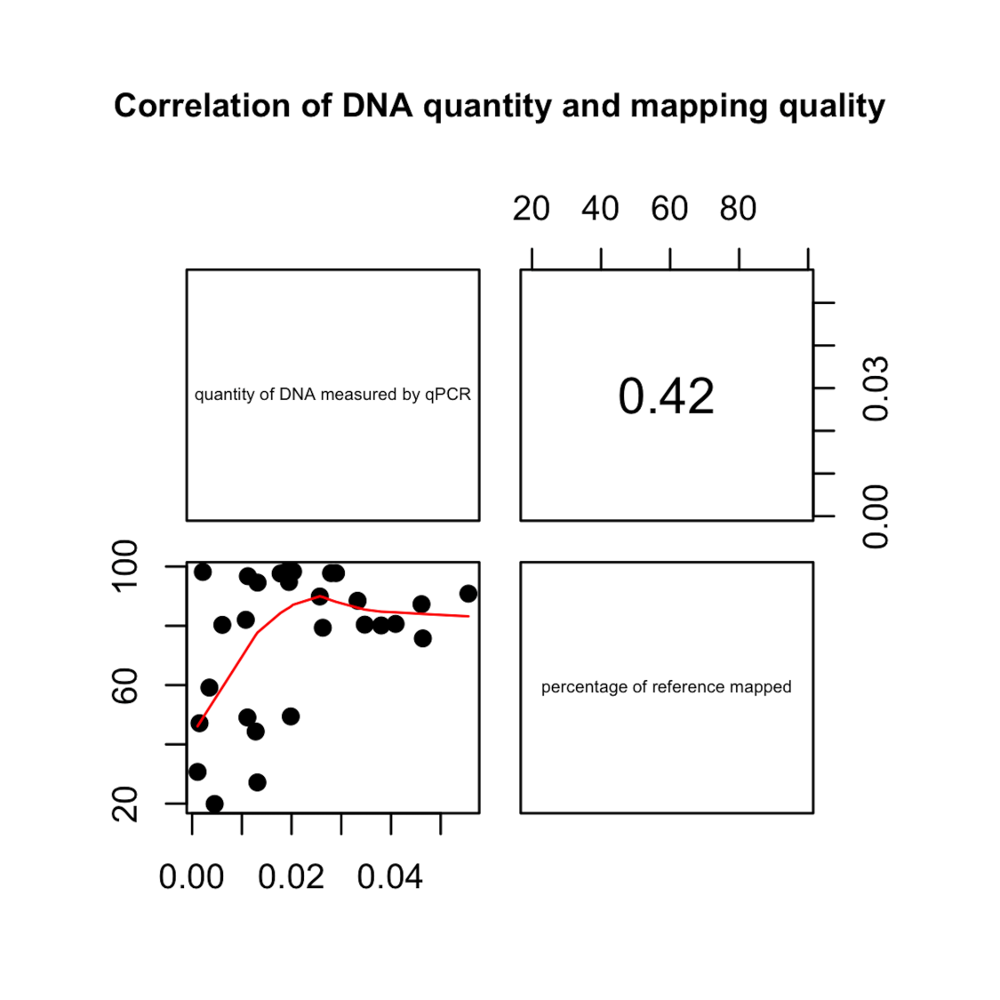

Supplement: S2 Fig — Positive Spearman correlation between the quantity of Vibrio cholerae DNA in the material recovered from the Whatman 903 filter cards and the mapping quality as measured by percentage of Vibrio cholerae reference genome N16961 covered by short Illumina reads mapped by SMALT and mean depth of short Illumina reads. (TIF) [file pntd.0007330.s002.tif]

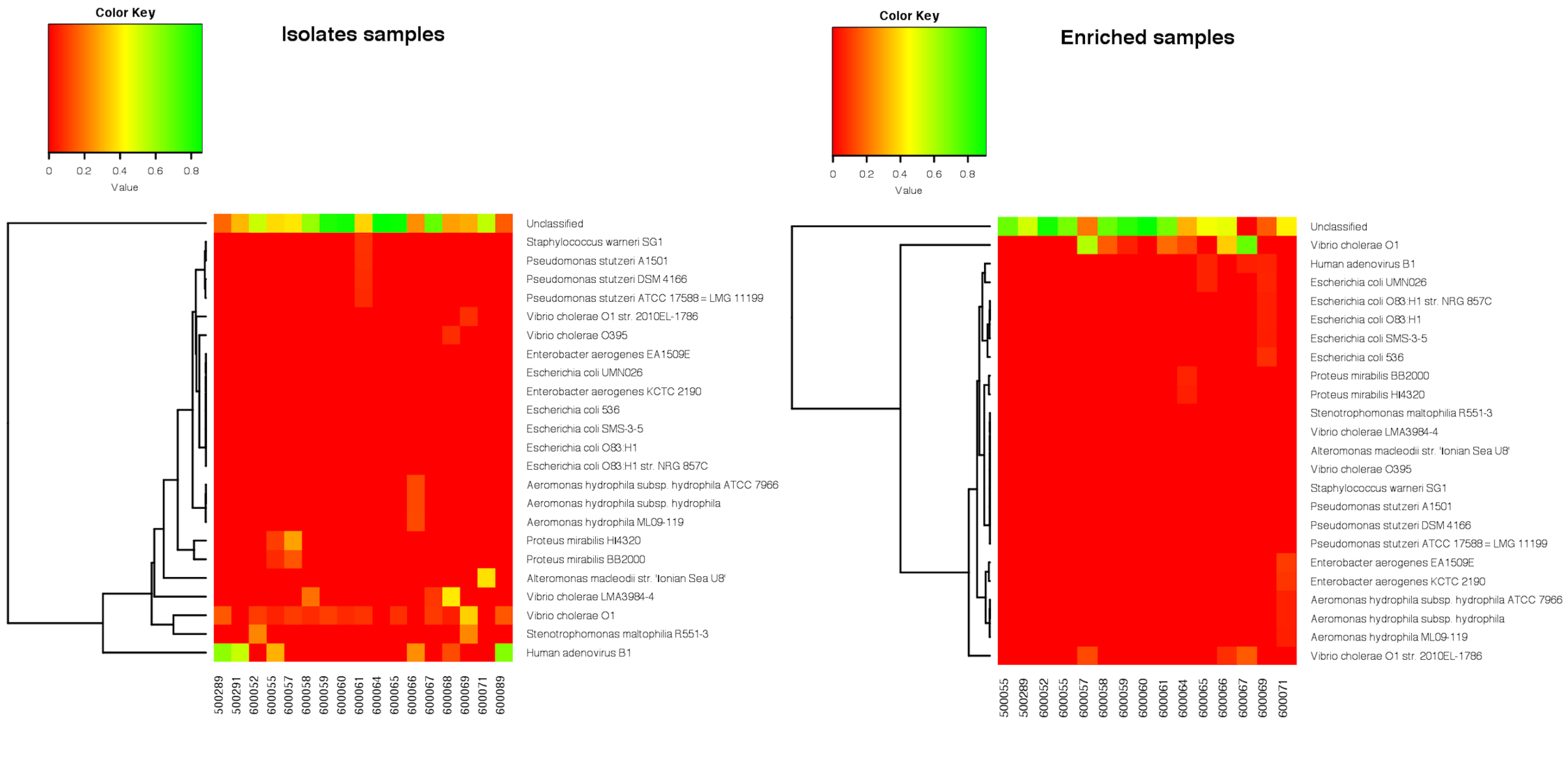

Supplement: S3 Fig — Proportion of reads specific to one species over all reads obtained from Kraken analysis of short read Illumina sequences of DNA recovered from Whatman 903 filter cards of APW-enriched specimen spotted filter papers versus culture isolates spotted filter papers (Selective threshold > 0.1%) (TIF) [file pntd.0007330.s003.tif]

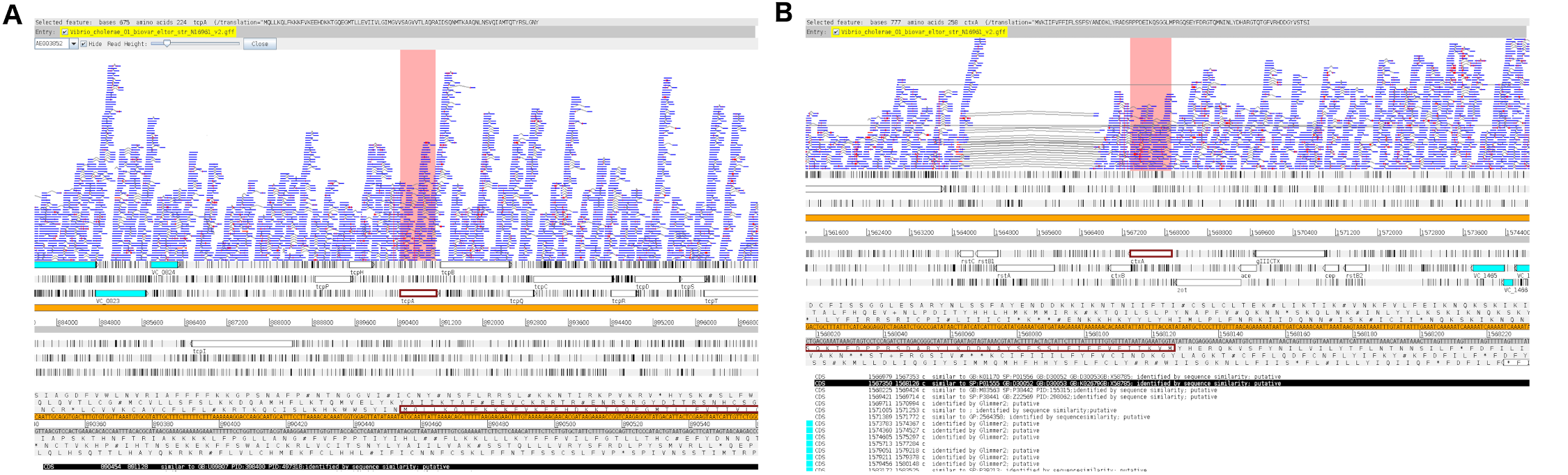

Supplement: S4 Fig — (TIF) [file pntd.0007330.s004.tif]

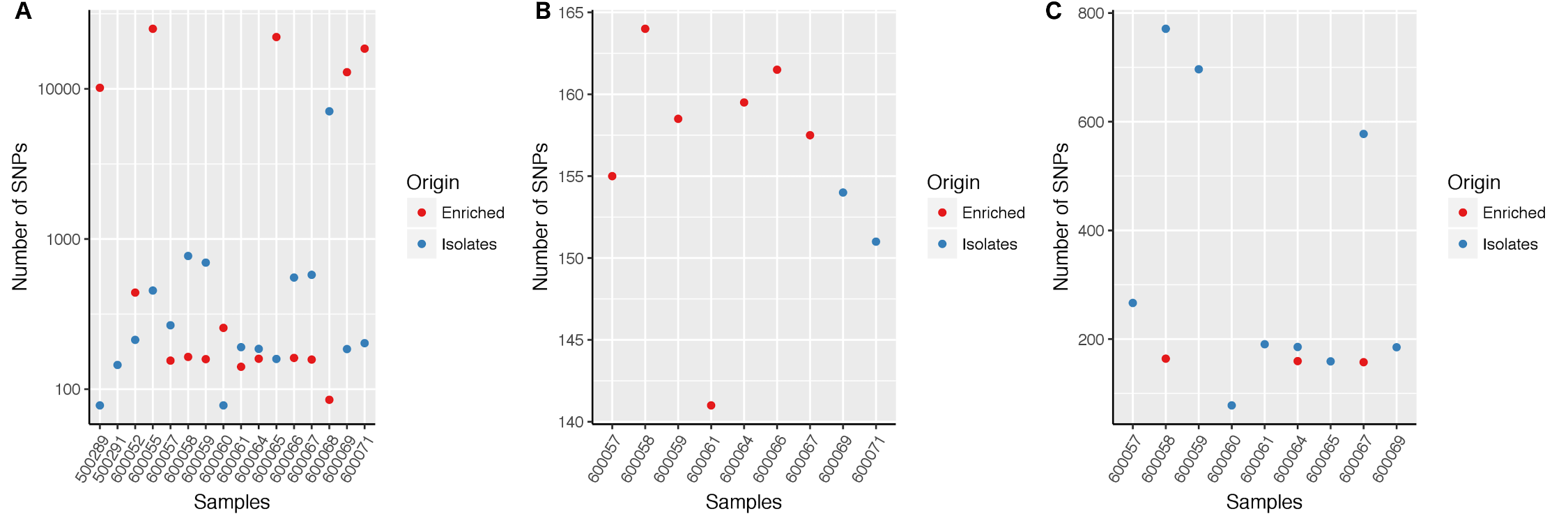

Supplement: S5 Fig — SNP calling based on SMALT mapping of short read Illumina sequences of DNA recovered from Whatman 903 filter cards of APW-enriched specimen versus culture isolate spotted filter papers samples (A). Comparison of SNP between APW-enriched specimen and culture isolate among samples with higher than 75% of Vibrio cholerae reference genome N16961 mapped(B) and among samples with higher than 50% of Vibrio cholerae reference genome N16961 mapped, higher than 0.02ng/μL Vibrio cholerae DNA and higher than 20x mean depth (C). (TIF) [file pntd.0007330.s005.tif]
