# Supplementary material for: Whole genome sequence of Vibrio cholerae directly from dried spotted filter paper
Source: PLoS Negl Trop Dis. 2019 May 30;13(5):e0007330. doi: 10.1371/journal.pntd.0007330 (PMC6559667; doi:10.1371/journal.pntd.0007330)
Supplement: S1 Table — (DOCX) [file pntd.0007330.s006.docx]

| **Sample.Name** | **Origin** | **Contig.length** | **Total.Reads** | **Mean.length** | **Mapped** | **Mapped.length** | **Proper.pairs** | **Unmapped** | **Chimeras** | **Errors** | **Insertions** | **Deletions** | **Errors.per.mapped.base** | **Insertions.per.mapped.base** | **Deletions.per.mapped.base** | **Mean.depth** | **Std.depth** | **Median.depth** | **Count.of.unmapped.bases.in.contig** | **X..ref.mapped** |
| --- | --- | --- | --- | --- | --- | --- | --- | --- | --- | --- | --- | --- | --- | --- | --- | --- | --- | --- | --- | --- |
| 600055_crude | Enriched | 4033464 | 20678058 | 100 | 1337594 | 57472618 | 63942 | 19340464 | 11666 | 7191382 | 93016 | 91964 | 0.13 | 0.00 | 0.00 | 14.25 | 43.94 | 0 | 2312915 | 42.66 |
| 600055_crude | Enriched | 4033464 | 20894446 | 100 | 1355916 | 58337336 | 65242 | 19538530 | 11941 | 7307741 | 94628 | 93527 | 0.13 | 0.00 | 0.00 | 14.46 | 44.52 | 0 | 2315021 | 42.60 |
| 600055_crude | Enriched | 4033464 | 47899548 | 100 | 3297483 | 145077039 | 172282 | 44602065 | 30248 | 18474845 | 239267 | 237616 | 0.13 | 0.00 | 0.00 | 35.97 | 111.20 | 0 | 2525954 | 37.38 |
| 600055_crude | Enriched | 4033464 | 48286410 | 100 | 3329321 | 146581162 | 173545 | 44957089 | 30417 | 18666569 | 242051 | 239594 | 0.13 | 0.00 | 0.00 | 36.34 | 112.39 | 0 | 2538589 | 37.06 |
| 600057_crude | Enriched | 4033464 | 9757534 | 100 | 8691755 | 850995715 | 4160982 | 1065779 | 44126 | 6053522 | 140301 | 110139 | 0.01 | 0.00 | 0.00 | 210.98 | 81.57 | 202 | 72208 | 98.21 |
| 600057_crude | Enriched | 4033464 | 9832268 | 100 | 8754038 | 857731032 | 4190583.5 | 1078230 | 45336 | 5803485 | 130117 | 104592 | 0.01 | 0.00 | 0.00 | 212.65 | 82.12 | 204 | 73675 | 98.17 |
| 600058_crude | Enriched | 4033464 | 22067908 | 100 | 13912726 | 1270241169 | 4149648.5 | 8155182 | 551058 | 10925087 | 271555 | 267499 | 0.01 | 0.00 | 0.00 | 314.93 | 122.72 | 305 | 68279 | 98.31 |
| 600058_crude | Enriched | 4033464 | 22293198 | 100 | 14052032 | 1283834898 | 4192585.5 | 8241166 | 555994 | 10648614 | 258175 | 260149 | 0.01 | 0.00 | 0.00 | 318.30 | 123.98 | 308 | 67186 | 98.33 |
| 600059_crude | Enriched | 4033464 | 27813730 | 100 | 11338839 | 994538504 | 3283381.5 | 16474891 | 303005 | 9232084 | 226068 | 228278 | 0.01 | 0.00 | 0.00 | 246.57 | 101.61 | 237 | 70650 | 98.25 |
| 600059_crude | Enriched | 4033464 | 28162668 | 100 | 11494914 | 1008767936 | 3329659.5 | 16667754 | 307262 | 9077450 | 218876 | 225569 | 0.01 | 0.00 | 0.00 | 250.10 | 102.70 | 240 | 69474 | 98.28 |
| 600060_crude | Enriched | 4033464 | 17885184 | 100 | 3117916 | 276780432 | 1237310 | 14767268 | 20345 | 2574604 | 62175 | 51713 | 0.01 | 0.00 | 0.00 | 68.62 | 49.93 | 61 | 217472 | 94.61 |
| 600060_crude | Enriched | 4033464 | 18105420 | 100 | 3155270 | 280252270 | 1252279.5 | 14950150 | 20595 | 2527529 | 60471 | 50442 | 0.01 | 0.00 | 0.00 | 69.48 | 50.52 | 62 | 220625 | 94.53 |
| 600061_crude | Enriched | 4033464 | 12590296 | 100 | 9092159 | 885873015 | 4352097.5 | 3498137 | 33338 | 6236396 | 143724 | 112497 | 0.01 | 0.00 | 0.00 | 219.63 | 118.40 | 208 | 93772 | 97.68 |
| 600061_crude | Enriched | 4033464 | 12725990 | 100 | 9188429 | 895851624 | 4398675 | 3537561 | 33910 | 6038170 | 133985 | 107716 | 0.01 | 0.00 | 0.00 | 222.10 | 119.59 | 210 | 95338 | 97.64 |
| 600064_crude | Enriched | 4033464 | 2867930 | 100 | 956100 | 84298603 | 389003 | 1911830 | 4708 | 1600759 | 25723 | 25451 | 0.02 | 0.00 | 0.00 | 20.90 | 15.97 | 19 | 89229 | 97.79 |
| 600064_crude | Enriched | 4033464 | 2891458 | 100 | 965874 | 85261496 | 393278.5 | 1925584 | 4865 | 1601479 | 25125 | 25148 | 0.02 | 0.00 | 0.00 | 21.14 | 16.03 | 19 | 89526 | 97.78 |
| 600065_crude | Enriched | 4033464 | 9091692 | 100 | 871768 | 44682057 | 135427 | 8219924 | 7143 | 5134566 | 60436 | 60152 | 0.11 | 0.00 | 0.00 | 11.08 | 45.34 | 0 | 2711439 | 32.78 |
| 600065_crude | Enriched | 4033464 | 9149532 | 100 | 880249 | 45195975 | 136964 | 8269283 | 7335 | 5186787 | 61300 | 61296 | 0.11 | 0.00 | 0.00 | 11.21 | 46.05 | 0 | 2708715 | 32.84 |
| 600065_crude | Enriched | 4033464 | 25828866 | 100 | 2560795 | 132440435 | 413088 | 23268071 | 21752 | 15505203 | 183457 | 183224 | 0.12 | 0.00 | 0.00 | 32.84 | 134.10 | 0 | 2888326 | 28.39 |
| 600065_crude | Enriched | 4033464 | 25555630 | 100 | 2529697 | 130906449 | 407988 | 23025933 | 21415 | 15315419 | 181532 | 180527 | 0.12 | 0.00 | 0.00 | 32.46 | 132.37 | 0 | 2873967 | 28.75 |
| 600066_crude | Enriched | 4033464 | 19560066 | 100 | 16357041 | 1611228866 | 8095171.5 | 3203025 | 4048 | 11236796 | 253800 | 191623 | 0.01 | 0.00 | 0.00 | 399.47 | 147.66 | 391 | 73963 | 98.17 |
| 600066_crude | Enriched | 4033464 | 19768306 | 100 | 16536416 | 1630003001 | 8184460 | 3231890 | 3818 | 10857319 | 237577 | 183144 | 0.01 | 0.00 | 0.00 | 404.12 | 149.38 | 395 | 73114 | 98.19 |
| 600067_crude | Enriched | 4033464 | 12226558 | 100 | 11380586 | 1120871450 | 5599524 | 845972 | 22410 | 7322997 | 174350 | 130279 | 0.01 | 0.00 | 0.00 | 277.89 | 121.89 | 267 | 89936 | 97.77 |
| 600067_crude | Enriched | 4033464 | 12315082 | 100 | 11466258 | 1130116966 | 5641869 | 848824 | 22485 | 7022733 | 160516 | 122597 | 0.01 | 0.00 | 0.00 | 280.19 | 122.37 | 269 | 89021 | 97.79 |
| 600068_crude | Enriched | 4033464 | 12779722 | 100 | 9282179 | 909202442 | 4538750.5 | 3497543 | 16810 | 6050746 | 144188 | 104650 | 0.01 | 0.00 | 0.00 | 225.41 | 350.17 | 0 | 2151015 | 46.67 |
| 600068_crude | Enriched | 4033464 | 12891206 | 100 | 9363431 | 917789370 | 4578374 | 3527775 | 17056 | 5816691 | 134605 | 99334 | 0.01 | 0.00 | 0.00 | 227.54 | 353.45 | 0 | 2150334 | 46.69 |
| 600068_crude | Enriched | 4033464 | 29051294 | 100 | 20378194 | 2012911026 | 9967659 | 8673100 | 37728 | 2513351 | 68842 | 64555 | 0.00 | 0.00 | 0.00 | 499.05 | 769.27 | 1 | 1933425 | 52.07 |
| 600068_crude | Enriched | 4033464 | 29154626 | 100 | 20453443 | 2020501771 | 10006533.5 | 8701183 | 37435 | 2432690 | 68923 | 65123 | 0.00 | 0.00 | 0.00 | 500.93 | 772.10 | 1 | 1926833 | 52.23 |
| 600069_crude | Enriched | 4033464 | 13746658 | 100 | 1900468 | 125553091 | 483204.5 | 11846190 | 11300 | 8812750 | 108580 | 111814 | 0.07 | 0.00 | 0.00 | 31.13 | 69.88 | 7 | 1653459 | 59.01 |
| 600069_crude | Enriched | 4033464 | 13885846 | 100 | 1920990 | 127092808 | 489581 | 11964856 | 11494 | 8877692 | 109446 | 112344 | 0.07 | 0.00 | 0.00 | 31.51 | 70.65 | 7 | 1654036 | 58.99 |
| 600069_crude | Enriched | 4033464 | 79625624 | 100 | 11376616 | 754972797 | 2933054 | 68249008 | 69788 | 53023751 | 631957 | 666655 | 0.07 | 0.00 | 0.00 | 187.18 | 424.16 | 49 | 1646526 | 59.18 |
| 600069_crude | Enriched | 4033464 | 77108006 | 100 | 11016775 | 731364280 | 2841754 | 66091231 | 67735 | 51379199 | 609714 | 643733 | 0.07 | 0.00 | 0.00 | 181.32 | 410.54 | 47 | 1637545 | 59.40 |
| 600071_crude | Enriched | 4033464 | 24663478 | 100 | 2255211 | 124100929 | 383173 | 22408267 | 16055 | 10926367 | 142568 | 142851 | 0.09 | 0.00 | 0.00 | 30.77 | 62.07 | 12 | 788882 | 80.44 |
| 600071_crude | Enriched | 4033464 | 24867760 | 100 | 2274150 | 125196396 | 386832 | 22593610 | 16256 | 11013006 | 144828 | 144519 | 0.09 | 0.00 | 0.00 | 31.04 | 62.57 | 12 | 798083 | 80.21 |
| 500289_crude | Enriched | 4033464 | 20125518 | 100 | 2121092 | 125921725 | 383333 | 18004426 | 17774 | 9622089 | 122944 | 130754 | 0.08 | 0.00 | 0.00 | 31.22 | 77.60 | 1 | 1975384 | 51.03 |
| 500289_crude | Enriched | 4033464 | 20332214 | 100 | 2139157 | 126963005 | 386433 | 18193057 | 17764 | 9681663 | 124343 | 132086 | 0.08 | 0.00 | 0.00 | 31.48 | 78.68 | 1 | 1985997 | 50.76 |
| 500289_crude | Enriched | 4033464 | 29476246 | 100 | 3217298 | 191659464 | 586183 | 26258948 | 28983 | 14828765 | 185083 | 199849 | 0.08 | 0.00 | 0.00 | 47.52 | 118.48 | 0 | 2282307 | 43.42 |
| 500289_crude | Enriched | 4033464 | 29696192 | 100 | 3246447 | 193412601 | 591854 | 26449745 | 29393 | 14940674 | 186180 | 200524 | 0.08 | 0.00 | 0.00 | 47.95 | 119.55 | 0 | 2286749 | 43.31 |
| 500289_EtOH | Isolates | 4033464 | 8877196 | 100 | 5779608 | 550928445 | 2362350 | 3097588 | 123338 | 4372104 | 100310 | 92202 | 0.01 | 0.00 | 0.00 | 136.59 | 229.78 | 0 | 2098406 | 47.98 |
| 500289_EtOH | Isolates | 4033464 | 8951916 | 100 | 5826215 | 555875132 | 2384260.5 | 3125701 | 123676 | 4227603 | 95151 | 89812 | 0.01 | 0.00 | 0.00 | 137.82 | 231.74 | 0 | 2116410 | 47.53 |
| 500289_EtOH | Isolates | 4033464 | 25592928 | 100 | 14855756 | 1424814059 | 6052889 | 10737172 | 323313 | 3638237 | 88889 | 124284 | 0.00 | 0.00 | 0.00 | 353.25 | 572.12 | 1 | 2001814 | 50.37 |
| 500289_EtOH | Isolates | 4033464 | 25759656 | 100 | 14944208 | 1433467549 | 6085094 | 10815448 | 325908 | 3567083 | 89023 | 125398 | 0.00 | 0.00 | 0.00 | 355.39 | 575.89 | 1 | 2000223 | 50.41 |
| 500291_EtOH | Isolates | 4033464 | 20199780 | 100 | 5775553 | 527273237 | 2238361.5 | 14424227 | 124666 | 5856652 | 116588 | 119628 | 0.01 | 0.00 | 0.00 | 130.72 | 462.51 | 0 | 2830540 | 29.82 |
| 500291_EtOH | Isolates | 4033464 | 20338808 | 100 | 5822450 | 532127246 | 2258267 | 14516358 | 125437 | 5746561 | 110806 | 116883 | 0.01 | 0.00 | 0.00 | 131.93 | 466.71 | 0 | 2836347 | 29.68 |
| 500291_EtOH | Isolates | 4033464 | 80160794 | 100 | 769437 | 35524486 | 103594 | 79391357 | 6123 | 642330 | 10306 | 28808 | 0.02 | 0.00 | 0.00 | 8.81 | 297.27 | 0 | 3040848 | 24.61 |
| 500291_EtOH | Isolates | 4033464 | 81231620 | 100 | 799450 | 35911601 | 102383 | 80432170 | 6141 | 681174 | 10445 | 29907 | 0.02 | 0.00 | 0.00 | 8.90 | 329.37 | 0 | 3047654 | 24.44 |
| 600052_crude | Enriched | 4033464 | 11443500 | 100 | 2875911 | 237386614 | 757403.5 | 8567589 | 66175 | 3382366 | 71056 | 69046 | 0.01 | 0.00 | 0.00 | 58.85 | 46.23 | 54 | 130479 | 96.77 |
| 600052_crude | Enriched | 4033464 | 11546242 | 100 | 2896660 | 239082125 | 762894.5 | 8649582 | 66181 | 3348914 | 68906 | 68546 | 0.01 | 0.00 | 0.00 | 59.27 | 46.61 | 54 | 131508 | 96.74 |
| 600055_EtOH | Isolates | 4033464 | 13167824 | 100 | 5494995 | 478488246 | 1957057.5 | 7672829 | 85189 | 6981273 | 131340 | 144564 | 0.01 | 0.00 | 0.00 | 118.63 | 223.10 | 0 | 2202076 | 45.40 |
| 600055_EtOH | Isolates | 4033464 | 13280474 | 100 | 5543999 | 483076998 | 1976551 | 7736475 | 85096 | 6898634 | 126694 | 142944 | 0.01 | 0.00 | 0.00 | 119.77 | 225.25 | 0 | 2215897 | 45.06 |
| 600055_EtOH | Isolates | 4033464 | 22749394 | 100 | 8774182 | 769388269 | 3102363 | 13975212 | 143643 | 6950635 | 121598 | 171769 | 0.01 | 0.00 | 0.00 | 190.75 | 348.93 | 0 | 2276130 | 43.57 |
| 600055_EtOH | Isolates | 4033464 | 22884932 | 100 | 8834974 | 774874207 | 3121160.5 | 14049958 | 145521 | 6962113 | 122338 | 173510 | 0.01 | 0.00 | 0.00 | 192.11 | 351.65 | 0 | 2287058 | 43.30 |
| 600057_EtOH | Isolates | 4033464 | 4721530 | 100 | 2140992 | 194286937 | 702952 | 2580538 | 63192 | 2007463 | 23627 | 52571 | 0.01 | 0.00 | 0.00 | 48.17 | 49.40 | 38 | 591737 | 85.33 |
| 600057_EtOH | Isolates | 4033464 | 5354794 | 100 | 550399 | 41227549 | 155300.5 | 4804395 | 9633 | 536119 | 4894 | 11577 | 0.01 | 0.00 | 0.00 | 10.22 | 65.87 | 4 | 338747 | 91.60 |
| 600058_EtOH | Isolates | 4033464 | 5799706 | 100 | 3586781 | 341592376 | 1370239 | 2212925 | 92693 | 4061144 | 34323 | 71839 | 0.01 | 0.00 | 0.00 | 84.69 | 107.80 | 44 | 1367767 | 66.09 |
| 600058_EtOH | Isolates | 4033464 | 4683680 | 100 | 2134525 | 193625120 | 702709.5 | 2549155 | 62495 | 1970815 | 23742 | 52567 | 0.01 | 0.00 | 0.00 | 48.00 | 49.53 | 38 | 588574 | 85.41 |
| 600059_EtOH | Isolates | 4033464 | 7679566 | 100 | 4079091 | 386095543 | 1531735 | 3600475 | 113002 | 2463671 | 29220 | 66969 | 0.01 | 0.00 | 0.00 | 95.72 | 68.68 | 85 | 201635 | 95.00 |
| 600059_EtOH | Isolates | 4033464 | 5791704 | 100 | 3593106 | 342192660 | 1373213 | 2198598 | 93471 | 4054369 | 34905 | 72924 | 0.01 | 0.00 | 0.00 | 84.84 | 108.15 | 43 | 1359642 | 66.29 |
| 600060_EtOH | Isolates | 4033464 | 2017958 | 100 | 613055 | 56459552 | 199751.5 | 1404903 | 24122 | 384906 | 4758 | 13101 | 0.01 | 0.00 | 0.00 | 14.00 | 21.41 | 7 | 1379870 | 65.79 |
| 600060_EtOH | Isolates | 4033464 | 7589636 | 100 | 4063199 | 384811604 | 1531693.5 | 3526437 | 112426 | 2338109 | 28786 | 67018 | 0.01 | 0.00 | 0.00 | 95.40 | 68.54 | 85 | 201362 | 95.01 |
| 600061_EtOH | Isolates | 4033464 | 6584192 | 100 | 2860740 | 262878075 | 925675.5 | 3723452 | 105724 | 2325850 | 28555 | 56038 | 0.01 | 0.00 | 0.00 | 65.17 | 65.42 | 52 | 516279 | 87.20 |
| 600061_EtOH | Isolates | 4033464 | 6519712 | 100 | 2852588 | 262261240 | 925296 | 3667124 | 105384 | 2253872 | 28284 | 56023 | 0.01 | 0.00 | 0.00 | 65.02 | 65.43 | 52 | 508179 | 87.40 |
| 600064_EtOH | Isolates | 4033464 | 6665558 | 100 | 2086536 | 192926217 | 836414 | 4579022 | 31236 | 1368198 | 16167 | 32547 | 0.01 | 0.00 | 0.00 | 47.83 | 52.48 | 37 | 839972 | 79.17 |
| 600064_EtOH | Isolates | 4033464 | 6582090 | 100 | 2075112 | 191921822 | 833995 | 4506978 | 30911 | 1301627 | 16082 | 32930 | 0.01 | 0.00 | 0.00 | 47.58 | 52.30 | 36 | 825897 | 79.52 |
| 600065_EtOH | Isolates | 4033464 | 6714598 | 100 | 3008472 | 283824948 | 1137453.5 | 3706126 | 79068 | 1856943 | 20776 | 49425 | 0.01 | 0.00 | 0.00 | 70.37 | 62.77 | 58 | 409376 | 89.85 |
| 600065_EtOH | Isolates | 4033464 | 6684326 | 100 | 3007412 | 283787944 | 1140283.5 | 3676914 | 79037 | 1777558 | 20797 | 49501 | 0.01 | 0.00 | 0.00 | 70.36 | 62.86 | 58 | 405714 | 89.94 |
| 600066_EtOH | Isolates | 4033464 | 10018466 | 100 | 1529484 | 113933625 | 436339 | 8488982 | 10523 | 3882954 | 52814 | 57476 | 0.03 | 0.00 | 0.00 | 28.25 | 121.40 | 0 | 3225430 | 20.03 |
| 600066_EtOH | Isolates | 4033464 | 10080260 | 100 | 1538014 | 114684506 | 438827 | 8542246 | 10378 | 3895940 | 52157 | 57608 | 0.03 | 0.00 | 0.00 | 28.43 | 122.09 | 0 | 3238090 | 19.72 |
| 600067_EtOH | Isolates | 4033464 | 9312044 | 100 | 5370881 | 507297068 | 1897128.5 | 3941163 | 178939 | 4903676 | 47662 | 102176 | 0.01 | 0.00 | 0.00 | 125.77 | 137.17 | 96 | 801430 | 80.13 |
| 600067_EtOH | Isolates | 4033464 | 9211322 | 100 | 5341859 | 504828840 | 1892705 | 3869463 | 178120 | 4730574 | 47078 | 101222 | 0.01 | 0.00 | 0.00 | 125.16 | 136.66 | 96 | 801967 | 80.12 |
| 600068_EtOH | Isolates | 4033464 | 8786982 | 100 | 6476391 | 605412962 | 2531022 | 2310591 | 149581 | 12139483 | 151715 | 141817 | 0.02 | 0.00 | 0.00 | 150.10 | 144.66 | 119 | 724201 | 82.05 |
| 600068_EtOH | Isolates | 4033464 | 8863102 | 100 | 6536274 | 611534639 | 2554257 | 2326828 | 151642 | 12072453 | 146069 | 139318 | 0.02 | 0.00 | 0.00 | 151.62 | 146.13 | 121 | 724857 | 82.03 |
| 600069_EtOH | Isolates | 4033464 | 8407588 | 100 | 6957730 | 665024815 | 2151135 | 1449858 | 386980 | 4294642 | 48800 | 141742 | 0.01 | 0.00 | 0.00 | 164.88 | 62.27 | 162 | 69310 | 98.28 |
| 600069_EtOH | Isolates | 4033464 | 10623588 | 100 | 5641486 | 529395602 | 2001784 | 4982102 | 182460 | 3667738 | 44547 | 125525 | 0.01 | 0.00 | 0.00 | 131.25 | 139.93 | 102 | 667998 | 83.44 |
| 600071_EtOH | Isolates | 4033464 | 5750422 | 100 | 557880 | 41589979 | 156071 | 5192542 | 9400 | 563270 | 5102 | 11675 | 0.01 | 0.00 | 0.00 | 10.31 | 65.70 | 4 | 350971 | 91.30 |
| 600071_EtOH | Isolates | 4033464 | 8300802 | 100 | 6888982 | 658638804 | 2134834.5 | 1411820 | 382239 | 4051822 | 47770 | 141247 | 0.01 | 0.00 | 0.00 | 163.29 | 61.76 | 161 | 70283 | 98.26 |
