# Supplementary material for: Whole genome sequence of Vibrio cholerae directly from dried spotted filter paper
Source: PLoS Negl Trop Dis. 2019 May 30;13(5):e0007330. doi: 10.1371/journal.pntd.0007330 (PMC6559667; doi:10.1371/journal.pntd.0007330)

**Supplementary Table 2:** Quast Spade assembly statistics of short Illumina reads obtained from sequencing of DNA recovered from APW-enriched specimen and culture isolates spotted Whatman 903 filter papers.

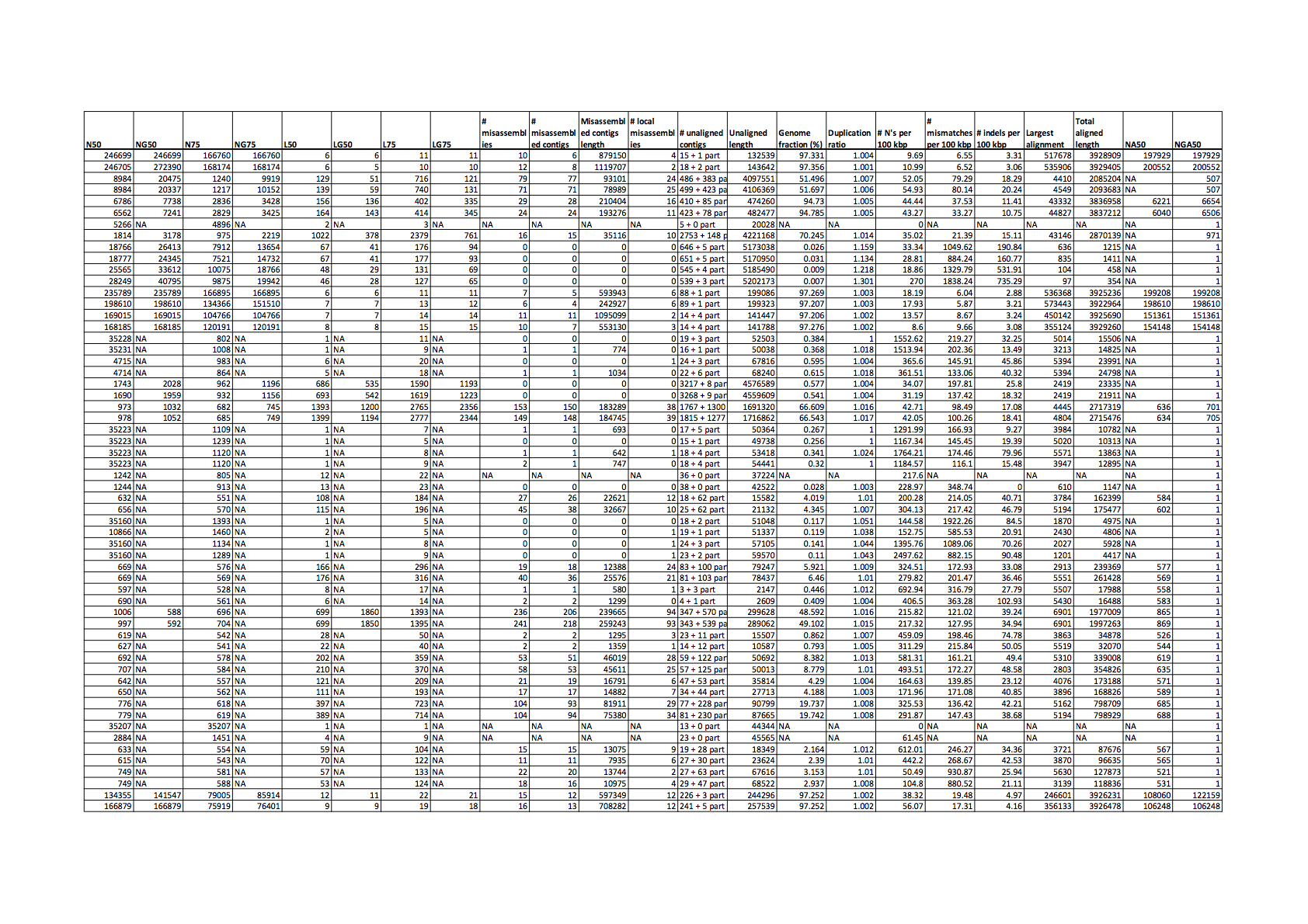


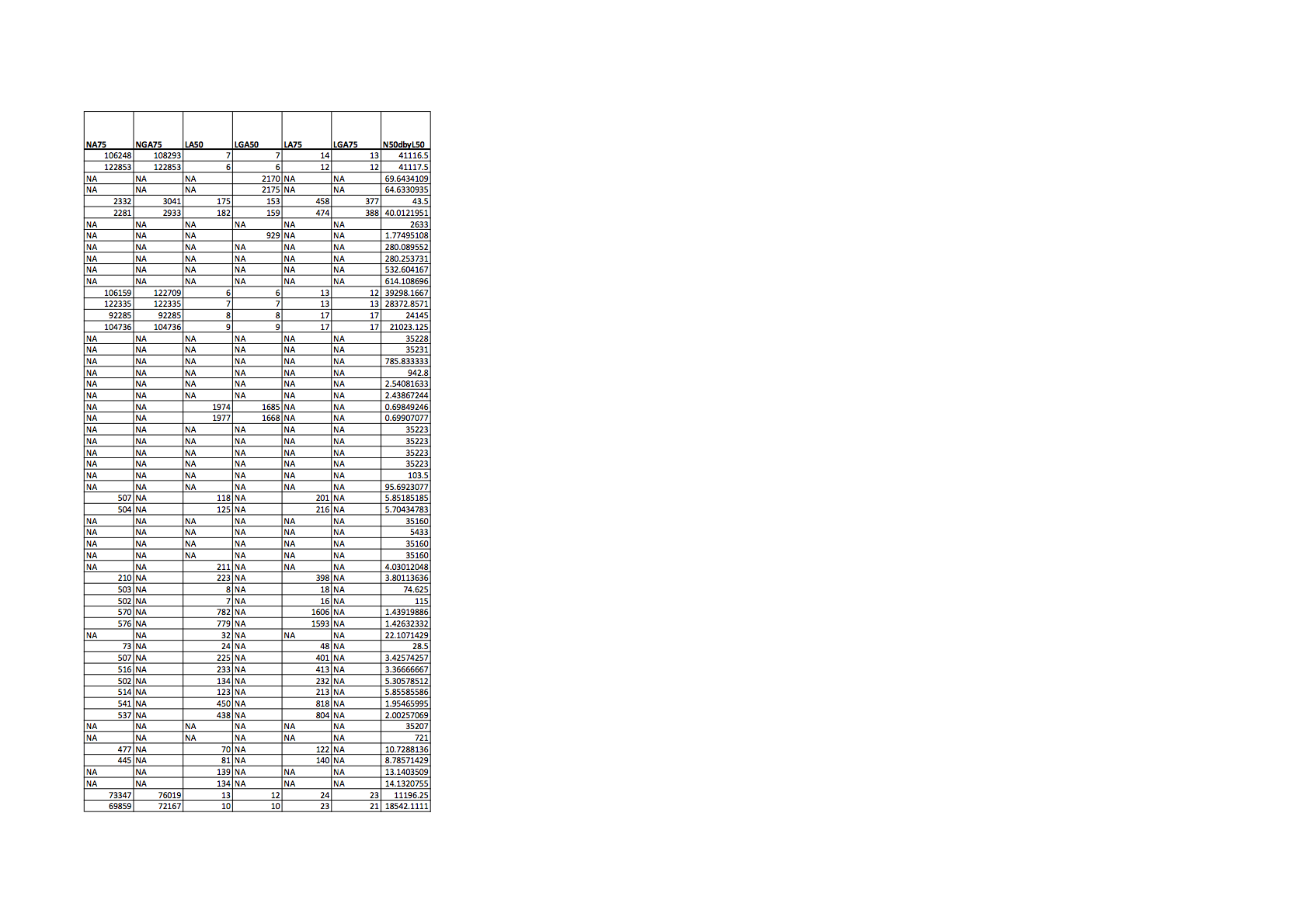

Supplement: S2 Table — (DOCX) [file pntd.0007330.s007.docx]
