# Supplementary material for: Whole genome sequence of Vibrio cholerae directly from dried spotted filter paper
Source: PLoS Negl Trop Dis. 2019 May 30;13(5):e0007330. doi: 10.1371/journal.pntd.0007330 (PMC6559667; doi:10.1371/journal.pntd.0007330)
Supplement: S3 Table — All data is available at https://www.ebi.ac.uk/ena. (DOCX) [file pntd.0007330.s008.docx]

| **Lane** | **Samples** | **Runs** | **Experiments** | **URL** |
| --- | --- | --- | --- | --- |
| 20613_1#1 | ERS2036365 | ERR2748972 | ERX2762026 | <https://www.ebi.ac.uk/ena/data/view/ERS2036365> |
| 20613_1#10 | ERS2036332 | ERR2748981 | ERX2762035 | <https://www.ebi.ac.uk/ena/data/view/ERS2036332> |
| 20613_1#11 | ERS2036333 | ERR2748982 | ERX2762036 | <https://www.ebi.ac.uk/ena/data/view/ERS2036333> |
| 20613_1#12 | ERS2036334 | ERR2748983 | ERX2762037 | <https://www.ebi.ac.uk/ena/data/view/ERS2036334> |
| 20613_1#13 | ERS2036335 | ERR2748984 | ERX2762038 | <https://www.ebi.ac.uk/ena/data/view/ERS2036335> |
| 20613_1#14 | ERS2036336 | ERR2748985 | ERX2762039 | <https://www.ebi.ac.uk/ena/data/view/ERS2036336> |
| 20613_1#15 | ERS2036337 | ERR2748986 | ERX2762040 | <https://www.ebi.ac.uk/ena/data/view/ERS2036337> |
| 20613_1#16 | ERS2036338 | ERR2748987 | ERX2762041 | <https://www.ebi.ac.uk/ena/data/view/ERS2036338> |
| 20613_1#17 | ERS2036339 | ERR2748988 | ERX2762042 | <https://www.ebi.ac.uk/ena/data/view/ERS2036339> |
| 20613_1#18 | ERS2036340 | ERR2748989 | ERX2762043 | <https://www.ebi.ac.uk/ena/data/view/ERS2036340> |
| 20613_1#19 | ERS2036341 | ERR2748990 | ERX2762044 | <https://www.ebi.ac.uk/ena/data/view/ERS2036341> |
| 20613_1#20 | ERS2036342 | ERR2748991 | ERX2762045 | <https://www.ebi.ac.uk/ena/data/view/ERS2036342> |
| 20613_1#21 | ERS2036343 | ERR2748992 | ERX2762046 | <https://www.ebi.ac.uk/ena/data/view/ERS2036343> |
| 20613_1#3 | ERS2036325 | ERR2748974 | ERX2762028 | <https://www.ebi.ac.uk/ena/data/view/ERS2036325> |
| 20613_1#4 | ERS2036326 | ERR2748975 | ERX2762029 | <https://www.ebi.ac.uk/ena/data/view/ERS2036326> |
| 20613_1#5 | ERS2036327 | ERR2748976 | ERX2762030 | <https://www.ebi.ac.uk/ena/data/view/ERS2036327> |
| 20613_1#6 | ERS2036328 | ERR2748977 | ERX2762031 | <https://www.ebi.ac.uk/ena/data/view/ERS2036328> |
| 20613_1#7 | ERS2036329 | ERR2748978 | ERX2762032 | <https://www.ebi.ac.uk/ena/data/view/ERS2036329> |
| 20613_1#8 | ERS2036330 | ERR2748979 | ERX2762033 | <https://www.ebi.ac.uk/ena/data/view/ERS2036330> |
| 20613_1#9 | ERS2036331 | ERR2748980 | ERX2762034 | <https://www.ebi.ac.uk/ena/data/view/ERS2036331> |
| 20613_2#1 | ERS2036365 | ERR2749000 | ERX2762054 | <https://www.ebi.ac.uk/ena/data/view/ERS2036365> |
| 20613_2#10 | ERS2036332 | ERR2749009 | ERX2762063 | <https://www.ebi.ac.uk/ena/data/view/ERS2036332> |
| 20613_2#11 | ERS2036333 | ERR2749010 | ERX2762064 | <https://www.ebi.ac.uk/ena/data/view/ERS2036333> |
| 20613_2#12 | ERS2036334 | ERR2749011 | ERX2762065 | <https://www.ebi.ac.uk/ena/data/view/ERS2036334> |
| 20613_2#13 | ERS2036335 | ERR2749012 | ERX2762066 | <https://www.ebi.ac.uk/ena/data/view/ERS2036335> |
| 20613_2#14 | ERS2036336 | ERR2749013 | ERX2762067 | <https://www.ebi.ac.uk/ena/data/view/ERS2036336> |
| 20613_2#15 | ERS2036337 | ERR2749014 | ERX2762068 | <https://www.ebi.ac.uk/ena/data/view/ERS2036337> |
| 20613_2#16 | ERS2036338 | ERR2749015 | ERX2762069 | <https://www.ebi.ac.uk/ena/data/view/ERS2036338> |
| 20613_2#17 | ERS2036339 | ERR2749016 | ERX2762070 | <https://www.ebi.ac.uk/ena/data/view/ERS2036339> |
| 20613_2#18 | ERS2036340 | ERR2749017 | ERX2762071 | <https://www.ebi.ac.uk/ena/data/view/ERS2036340> |
| 20613_2#19 | ERS2036341 | ERR2749018 | ERX2762072 | <https://www.ebi.ac.uk/ena/data/view/ERS2036341> |
| 20613_2#20 | ERS2036342 | ERR2749019 | ERX2762073 | <https://www.ebi.ac.uk/ena/data/view/ERS2036342> |
| 20613_2#21 | ERS2036343 | ERR2749020 | ERX2762074 | <https://www.ebi.ac.uk/ena/data/view/ERS2036343> |
| 20613_2#3 | ERS2036325 | ERR2749002 | ERX2762056 | <https://www.ebi.ac.uk/ena/data/view/ERS2036325> |
| 20613_2#4 | ERS2036326 | ERR2749003 | ERX2762057 | <https://www.ebi.ac.uk/ena/data/view/ERS2036326> |
| 20613_2#5 | ERS2036327 | ERR2749004 | ERX2762058 | <https://www.ebi.ac.uk/ena/data/view/ERS2036327> |
| 20613_2#6 | ERS2036328 | ERR2749005 | ERX2762059 | <https://www.ebi.ac.uk/ena/data/view/ERS2036328> |
| 20613_2#7 | ERS2036329 | ERR2749006 | ERX2762060 | <https://www.ebi.ac.uk/ena/data/view/ERS2036329> |
| 20613_2#8 | ERS2036330 | ERR2749007 | ERX2762061 | <https://www.ebi.ac.uk/ena/data/view/ERS2036330> |
| 20613_2#9 | ERS2036331 | ERR2749008 | ERX2762062 | <https://www.ebi.ac.uk/ena/data/view/ERS2036331> |
| 20926_1#1 | ERS2036351 | ERR2749028 | ERX2762082 | <https://www.ebi.ac.uk/ena/data/view/ERS2036351> |
| 20926_1#10 | ERS2036360 | ERR2749037 | ERX2762091 | <https://www.ebi.ac.uk/ena/data/view/ERS2036360> |
| 20926_1#11 | ERS2036361 | ERR2749038 | ERX2762092 | <https://www.ebi.ac.uk/ena/data/view/ERS2036361> |
| 20926_1#2 | ERS2036352 | ERR2749029 | ERX2762083 | <https://www.ebi.ac.uk/ena/data/view/ERS2036352> |
| 20926_1#3 | ERS2036353 | ERR2749030 | ERX2762084 | <https://www.ebi.ac.uk/ena/data/view/ERS2036353> |
| 20926_1#4 | ERS2036354 | ERR2749031 | ERX2762085 | <https://www.ebi.ac.uk/ena/data/view/ERS2036354> |
| 20926_1#5 | ERS2036355 | ERR2749032 | ERX2762086 | <https://www.ebi.ac.uk/ena/data/view/ERS2036355> |
| 20926_1#6 | ERS2036356 | ERR2749033 | ERX2762087 | <https://www.ebi.ac.uk/ena/data/view/ERS2036356> |
| 20926_1#7 | ERS2036357 | ERR2749034 | ERX2762088 | <https://www.ebi.ac.uk/ena/data/view/ERS2036357> |
| 20926_1#8 | ERS2036358 | ERR2749035 | ERX2762089 | <https://www.ebi.ac.uk/ena/data/view/ERS2036358> |
| 20926_1#9 | ERS2036359 | ERR2749036 | ERX2762090 | <https://www.ebi.ac.uk/ena/data/view/ERS2036359> |
| 20926_2#1 | ERS2036351 | ERR2749039 | ERX2762093 | <https://www.ebi.ac.uk/ena/data/view/ERS2036351> |
| 20926_2#10 | ERS2036360 | ERR2749047 | ERX2762101 | <https://www.ebi.ac.uk/ena/data/view/ERS2036360> |
| 20926_2#11 | ERS2036361 | ERR2749048 | ERX2762102 | <https://www.ebi.ac.uk/ena/data/view/ERS2036361> |
| 20926_2#2 | ERS2036352 | ERR2749040 | ERX2762094 | <https://www.ebi.ac.uk/ena/data/view/ERS2036352> |
| 20926_2#3 | ERS2036353 | ERR2749041 | ERX2762095 | <https://www.ebi.ac.uk/ena/data/view/ERS2036353> |
| 20926_2#4 | ERS2036354 | ERR2749042 | ERX2762096 | <https://www.ebi.ac.uk/ena/data/view/ERS2036354> |
| 20926_2#5 | ERS2036355 | ERR2749043 | ERX2762097 | <https://www.ebi.ac.uk/ena/data/view/ERS2036355> |
| 20926_2#6 | ERS2036356 | ERR2749044 | ERX2762098 | <https://www.ebi.ac.uk/ena/data/view/ERS2036356> |
| 20926_2#7 | ERS2036357 | ERR2749045 | ERX2762099 | <https://www.ebi.ac.uk/ena/data/view/ERS2036357> |
| 20926_2#8 | ERS2036358 | ERR2749046 | ERX2762100 | <https://www.ebi.ac.uk/ena/data/view/ERS2036358> |
| 20926_2#9 | ERS2036359 | ERR2752416 | ERX2765462 | <https://www.ebi.ac.uk/ena/data/view/ERS2036359> |
| 21576_1#1 | ERS2036337 | ERR2749049 | ERX2762103 | <https://www.ebi.ac.uk/ena/data/view/ERS2036337> |
| 21576_2#1 | ERS2036337 | ERR2749051 | ERX2762105 | <https://www.ebi.ac.uk/ena/data/view/ERS2036337> |
| 21590_1#1 | ERS2036365 | ERR2749053 | ERX2762107 | <https://www.ebi.ac.uk/ena/data/view/ERS2036365> |
| 21590_1#2 | ERS2036326 | ERR2749054 | ERX2762108 | <https://www.ebi.ac.uk/ena/data/view/ERS2036326> |
| 21590_1#3 | ERS2036336 | ERR2749055 | ERX2762109 | <https://www.ebi.ac.uk/ena/data/view/ERS2036336> |
| 21590_1#4 | ERS2036339 | ERR2749056 | ERX2762110 | <https://www.ebi.ac.uk/ena/data/view/ERS2036339> |
| 21590_1#5 | ERS2036341 | ERR2749057 | ERX2762111 | <https://www.ebi.ac.uk/ena/data/view/ERS2036341> |
| 21590_2#1 | ERS2036365 | ERR2749059 | ERX2762113 | <https://www.ebi.ac.uk/ena/data/view/ERS2036365> |
| 21590_2#2 | ERS2036326 | ERR2749060 | ERX2762114 | <https://www.ebi.ac.uk/ena/data/view/ERS2036326> |
| 21590_2#3 | ERS2036336 | ERR2749061 | ERX2762115 | <https://www.ebi.ac.uk/ena/data/view/ERS2036336> |
| 21590_2#4 | ERS2036339 | ERR2749062 | ERX2762116 | <https://www.ebi.ac.uk/ena/data/view/ERS2036339> |
| 21590_2#5 | ERS2036341 | ERR2749063 | ERX2762117 | <https://www.ebi.ac.uk/ena/data/view/ERS2036341> |
| 21591_1#1 | ERS2036333 | ERR2749065 | ERX2762119 | <https://www.ebi.ac.uk/ena/data/view/ERS2036333> |
| 21591_1#2 | ERS2036340 | ERR2749066 | ERX2762120 | <https://www.ebi.ac.uk/ena/data/view/ERS2036340> |
| 21591_2#1 | ERS2036333 | ERR2749070 | ERX2762124 | <https://www.ebi.ac.uk/ena/data/view/ERS2036333> |
| 21591_2#2 | ERS2036340 | ERR2749071 | ERX2762125 | <https://www.ebi.ac.uk/ena/data/view/ERS2036340> |
